# 

**Published:** 1890-06-14

**Authors:** 


### SATURDAY, JUNE 14th, 1890.

**The Prevention of Orphanhood** - - - - - 145
**Words of Consolation.**—Reading the Scriptures - - - 146
**The Doctor's Pulpit.**—A Physician's Apologia - - - 147
**The History and Capabilities of Herbal Simples.**—By W. T. Fernie, M.D. XI.—White Byrony - - - 148
**Before the Lords' Committee** (By our Special Commissioner). III.—Mr. Timothy Holmes, Mr. Nelson Hardy, and Mr. Hugh Woods - - - - - - 149
**Twelve English Statesmen.**—William I., Henry II., and Henry VII. - - - - - - 150
**Everybody's Page** - - - - - - - 151
**Notes and News** - - - - - - - 152
**Glances at Foreign Asylums** - - - - - 153
**Annotations.**—The British Medical Benevolent Fund—Board and Residence for a Lady—A Girl of Quality - - - 154
**The Practitioner's Mirror and Retrospect:**
Medicine—Phosphorus in Rickets—A New Antidote for Morphine—Atropine in Poisoning by Aconite—Indirect Mortality from Influenza, 155; Antisepsine—Diuretin - 156
Surgery—Temporary Resection of the Skull—Treatment of Traumatic Tetanus by Subcutaneous Injection of Carbolic Acid—Treatment of Varicose Veins by Enucleation - 156
Therapeutics—Salicylates, 156; Diet in Gout—Calomel in Cardiac Dropsy, 157; Paracresotinic Acid - - - 158
New Drugs, Appliances, and Things Medical—Rizine—The Patent Steel Wire Dominion Spring Mattress, and the "Lawson Tait" Bedstead—Benger's Preparations— 158
**June** - - - - - - - - - 159
**Passing Topics**—When Ought Appeals to Cease? - - 160
**Scraps and Gleanings** - - - - - - - 160

### EXTRA SUPPLEMENT—THE NURSING MIRROR.

**En Passant.**—One of the Elite—The Princess of Wales and the First Thousand—University College Hospital Bazaar—Insomnia—District Nursing at Manchester—To Asylum Attendants—A Summer Sermon - - - - xli
**Asylum Attendants.—Their Work and Training.** By Francis H. Walmsley, M.D. - - - - - - xlii
**Our Bird Cage** - - - - - - - - xliii
**Everybody's Opinion.**—The Registration of Midwives—A Home of Rest—"The Anæmic Look"—The Junius S. Morgan Memorial—Asylum Attendants - - - xliii
**Examination Questions** - - - - - - xliv
**A Nurse's Work** - - - - - - - xliv
**Notes and Queries—Vacancies** - - - - xliv
**Amusements and Relaxation** - - - - - xliv

